# Antioxidant and hypolipidemic activity of Kumbhajatu in hypercholesterolemic rats

**DOI:** 10.4103/0974-7788.72487

**Published:** 2010

**Authors:** Rumi Ghosh, Parag P. Kadam, Vilasrao J. Kadam

**Affiliations:** *Department of Pharmacology, Bharati Vidyapeeth’s College of Pharmacy, CBD, Belapur, Navi Mumbai, India*

**Keywords:** Antioxidant, *in vivo*, Kumbhajatu, lipids, simvastatin

## Abstract

**Objective:**

To study the efficacy of Kumbhajatu in reducing the cholesterol levels and as an antioxidant in hypercholesterolemic rats.

**Materials and Methods:**

Hypercholesterolemia was induced in normal rats by including 2% w/w cholesterol, 1% w/w sodium cholate and 2.5% w/w coconut oil in the normal diet. Powdered form of Kumbhajatu was administered as feed supplement at 250 and 500 mg/kg dose levels to the hypercholesterolemic rats. Plasma lipid profile, hepatic superoxide dismutase (SOD) activity, catalase activity, reduced glutathione and extent of lipid peroxidation in the form of malondialdehyde were estimated using standard methods.

**Results:**

Feed supplementation with 250 and 500 mg/kg of Kumbhajatu resulted in a significant decline in plasma lipid profiles. The feed supplementation increased the concentration of catalase, SOD, glutathione and HDL-c significantly in both the experimental groups (250 and 500 mg/kg). On the other hand, the concentration of malondialdehyde, cholesterol, triglycerides, LDL-c and VLDL in these groups (250 and 500 mg/kg) were decreased significantly.

**Conclusion:**

The present study demonstrates that addition of Kumbhajatu powder at 250 and 500 mg/kg level as a feed supplement reduces the plasma lipid levels and also decreases lipid peroxidation.

## INTRODUCTION

Raised serum lipid levels, particularly of cholesterol along with generation of Reactive Oxygen Species (ROS), play a key role in the development of Coronary Artery Disease (CAD) and atherosclerosis.[1] Coronary artery disease is a serious medical problem that affects millions of people annually throughout the world. People who are predisposed to a combination of risk factors (dietary habits, genetic susceptibility, etc.) are more prone to develop atherosclerosis and CAD. Besides stress, sedentary habits such as use of tobacco and alcohol are reported to have an additive effect in contributing to the development of atherosclerosis and CAD.[2] Dietary modifications, physical exercise, abstinence from tobacco and alcohol and changes in lifestyle have been proposed to reduce the incidence of CAD and other cardiac maladies by the medical fraternity all over the world. Phytosterols and natural antioxidants have also been shown to be effective in reducing lipid profiles and also mitigate peroxidative modification of lipoproteins and atherosclerosis.[3]

Kumbhajatu is a proprietary and polyherbal formulation, which consists of four main ingredients, viz. Lodhra (*Symplocos racemosa*), Jatamansi (*Nardostychs Jatamansi*), Kumbhi (*Careya arborea*) and Shilajit (*Asphaltum*). The stem bark of Lodhra (*Symplocos racemosa*) has anti-inflammatory properties and is used in the treatment of menorrhagia and uterine disorders. The dried rhizome of Jatamansi (*Nardostychs Jatamansi*) is one of the best herbs used in the treatment of epilepsy, hepatitis.[4] The bark of Kumbhi (*Careya arborea*) is used as antioxidant.[5] Shilajit (*Asphaltum*) is widely used in the preparation of Ayurvedic medicines and is regarded as one of the most important ingredients in Ayurvedic system of medicine. It is used as an adaptogen.[6]

As there are been no reports available on the hypocholesterolemic and antioxidant effect of Kumbhajatu, the present study was undertaken to evaluate its ability to reduce its cholesterol profile and to scavenge the free radicals.

## MATERIALS AND METHODS

### Polyherbal formulation

Kumbhajatu is manufactured by Ayurveda Rasashala, Pune, India. The composition is as follows: Shilajit (Asphaltum) 80 mg; Lodhra (*S. racemosa*) 60 mg; Jatamansi (*N. jatamansi*) 40 mg; Kumbhi (*C. arborea*) 45 mg; Loha Bhasma 20 mg; Suvarna sutshekhar 5 mg.

### Dose preparation

The powdered form of ‘Kumbhajatu’ was suspended in distilled water with 5% w/w gum acacia as a suspending agent. It was administered orally to the animals by gastric intubation.

### Chemicals

Cholesterol, sodium cholate, coconut oil, trichloroacetic acid, thiobarbituric acid, sodium dodecyl sulfate, phosphate buffer (pH 7.4), acetic acid, butanol, pyridine, EDTA, Ellman’s reagent (5,5’-dithiobis-2-nitrobenzoic acid), sodium citrate, sodium pyrophosphate buffer (pH 8.3, 0.052 M), Phenazine methosulfate, nitroblue tetrazolium, NADH, glacial acetic acid, phosphate buffer (pH 7), Hydrogen peroxide; all chemicals used including solvents were of analytical grade.

### Experimental design

Acute oral toxicity study was performed using Swiss albino female mice as per OECD (Organization for Economic Co-operation and Development) guidelines. Kumbhajatu was found to be safe up to 2000 mg/kg body weight when administered orally. Two doses of Kumbhajatu were selected for the study: 250 and 500 mg/kg.

The study was carried out after obtaining the Institutional Animal Ethics Committee (IAEC) approval. Sprague Dawley (SD) rats (female), maintained at a 12 h light/dark cycle, were used for the study. Animals were housed under standard laboratory conditions, with free access to food (commercial rat cubes from Amrut Laboratory, Thane, India) and water, *ad libitum*. Hyperlipidemia was induced by feeding a high cholesterol diet (regular diet mixed with 2% w/w cholesterol, 1% w/w sodium cholate and 2.5% w/w coconut oil) to healthy rats for five days. Rats were divided into five groups containing six animals each; Group 1 received normal diet (normal); Group 2 received high cholesterol diet (control); Group 3 received Kumbhajatu 250 mg/kg, p.o.; Group 4 received Kumbhajatu 500 mg/kg p.o. and Group 5 received simvastatin 10 mg/kg i.p. for six days. At the end of the sixth day, food was withdrawn, and on the seventh day, fasting blood samples were collected by retro-orbital puncture technique in a coagulant-free vessel, and were kept at room temperature for 1 h. Samples were centrifuged at 4000–5000 rpm to separate serum, which was subjected for the estimation of lipid profile.[7] Immediately after sacrificing the animals, livers were separated, washed with pH 7.4 buffer, blotted with dry filter paper and liver weight was recorded. A part of the liver was minced and then homogenized in pH 7.4 buffer and was used for the estimation of lipid peroxidation and reduced glutathione. Another part of the liver was minced and then homogenized in pH 7 buffer and was used for the estimation of Superoxide dismutase.

### Lipid peroxidation (LPO) activity

The tissues were homogenized in 0.1 M phosphate buffer pH 7.4 with a Teflon glass homogenizer. Lipid peroxidation in this homogenate was determined by measuring the amounts of malondialdehyde (MDA) produced primarily. 0.2 ml of tissue homogenate, 0.2 ml of 8.1 % of sodium dodecyl sulfate (SDS), 1.5 ml of 20 % acetic acid and 1.5 ml of 8 % TBA were added. The volume of mixture was made up to 4 ml with distilled water and then heated at 95°C on water bath for 60 min using glass ball as a condenser. After incubation, tubes were cooled to room temperature and final volume was made to 5 ml in each tube. 5 ml of butanol: pyridine (15: 1) mixture was added and the contents were vortexed thoroughly for 2 min. After centrifugation at 3000 rpm for 10 min, the upper organic layer was taken and its O.D. read at 532 nm against an appropriate blank without the sample. The level of lipid peroxides were expressed as *n* moles of thiobarbituric acid reactive substances (TBARS)/mg protein using an extinction coefficient of 1.56 × 105 MI/cm.[8]

### Reduced glutathione (GSH) assay

To measure the GSH level, the tissue homogenate (in 0.1 M phosphate buffer pH 7.4) was taken. The homogenate was added with equal volume of 20% trichloroacetic acid (TCA) containing 1 mM EDTA to precipitate the tissue proteins. The mixture was allowed to stand for 5 min prior to centrifugation for 10 min at 200 rpm. The supernatant (200 µl) was then transferred to a new set of test tubes to which were added 1.8 ml of Ellman’s reagent (5,5'-dithiobis-2-nitrobenzoic acid) (0.1 mM) which was prepared in 0.3 M phosphate buffer with 1% of sodium citrate solution. Then all the test tubes made up to the volume of 2 ml. After completion of the total reaction, solutions were measured at 412 nm against blank. Absorbance values were compared with a standard curve generated from standard curve from known GSH.[8]

### Superoxide dismutase (SOD) assay

Assay mixture contained 0.1 ml of sample. A 1.2 ml of sodium pyrophosphate buffer (pH 8.3, 0.052 M), 0.1 ml of Phenazine methosulphate (186 µM), 0.3 ml of 300 µM nitroblue tetrazolium, 0.2 ml NADH. After incubation at 30°C for 90 s, the reaction was stopped by the addition of 0.1 ml glacial acetic acid. Reaction mixture was stirred vigorously with 4 ml of *n*-butanol. Mixture was allowed to stand for 10 min, centrifuged and butanol layer was separated. Color intensity of the chromogen in the butanol layer was measured at 560 nm spectrophotometrically and concentration of SOD was expressed as units/mg protein.[8]

### Catalase (CAT) assay

A 0.1 ml of supernatant was added to cuvette containing 1.9 ml of 50 mM phosphate buffer (pH 7). Reaction was started by the addition of 1 ml of freshly prepared 30 mM H_2_O_2_. The rate of decomposition of H_2_O_2_ was measured spectrophotometrically from changes in absorbance at 240 nm. Activity of catalase was expressed as unit/mg protein.[8]

### Statistical analysis

All data are presented as mean ± SEM. To investigate the relationship among the groups, one-way ANOVA followed by Dunnett’s test, was performed using Graph Pad Prism, Version 5.0 (Graph Pad Software, San Diego, CA, USA). *P*-values <0.05 were considered significant.

## RESULTS

**Figure 1:**
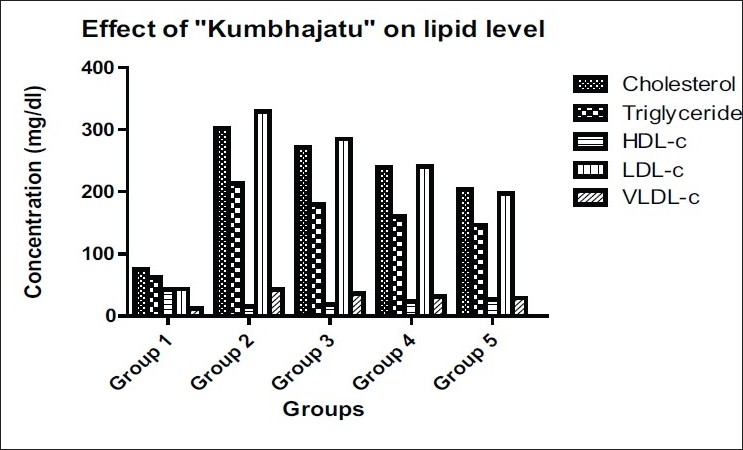
Effect of Kumbhajatu on lipid levels

Addition of Kumbhajatu as a feed supplement at two doses, i.e. 250 and 500 mg/kg, resulted in a dose-dependent reduction in lipid profiles in plasma along with significant reduction in lipid peroxidation. Figure 1 and Table 1 shows that, the total lipids i.e. total cholesterol and triglycerides in plasma as well as LDL and VLDL cholesterol were significantly reduced at both doses of feed supplementation. However, HDL-cholesterol level increased in both treated groups significantly. This observation indicates that Kumbhajatu, as a feed component is effective in reducing plasma LDL and VLDL-c levels. It is well known that increased HDL-c levels have a protective role in CAD.

**Table 1:** Effect of Kumbhajatu on lipid profile

Groups	Parameter				
	TC (mg/dl)	TG (mg/dl)	LDL-c (mg/kg)	HDL-c (mg/dl)	VLDL-c (mg/dl)
Group 1	74.88 ± 1.84	62.57 ± 0.84	43.93 ± 1.25	43.35 ± 1.07	12.40 ± 0.17
Group 2	302.04 ± 1.42^a^	213.81 ± 1.68^a^	329.27 ± 1.63^a^	15.64 ± 0.48^a^	42.75 ± 0.33^a^
Group 3	271.96 ± 1.44^b^	180.33 ± 0.76^c^	284.92 ± 1.42^c^	18.49 ± 0.30^c^	36.06 ± 0.15^b^
Group 4	238.87 ± 0.97^d^	160.49 ± 0.78^c^	240.74 ± 1.67^c^	23.10 ± 0.30^c^	32.09 ± 0.15^b^
Group 5	204.02 ± 1.94^d^	146.46 ± 2.97^d^	197.49 ± 2.37^d^	26.49 ± 0.16^d^	29.28 ± 0.59^d^

TC – Total Cholesterol, TG – Triglycerides, LDL-c – Low density lipoprotein, HDL-c – High density lipoprotein, VLDL-c – Very low density lipoprotein. n = 6 animals in each group. Values are expressed as mean ± SEM. Unpaired ‘t’ test when compared with vehicle control

a *P* < 0.001. One way ANOVA followed by Dunnett’s test when compared with Group 2

b *P* < 0.05,

c *P* < 0.01,

d *p* < 0.001.

Table 2 shows that the Kumbhajatu-treated groups have higher levels of antioxidative parameters (catalase, superoxide dismutase and glutathione) and decreased level of lipid peroxidation indicating its efficacy to reduce the LDL-c oxidation. Standard drug simvastatin, at a dose of 10 mg/kg of body wt. cause decreased serum cholesterol, triglyceride, LDL-c and VLDL-c levels, whereas HDL-cholesterol was increased more as compared to both doses of the test drug.

**Table 2:** Effect of Kumbhajatu on antioxidant parameters

Groups	Parameter			
	LPO (nMol /g wt)	CAT (mM of H_2_O_2_ /min/ g wt)	SOD (U/mg /min)	GSH (m mole/g of tissue)
Group 1	21.67 ± 0.61	52.33 ± 0.86	5.31 ± 0.18	34.76 ± 0.29
Group 2	38.74 ± 0.47^a^	30.57 ± 0.60^a^	2.39 ± 0.76^a^	13.39 ± 0.70^a^
Group 3	33.47 ± 0.28^c^	42.27 ± 0.54^c^	2.83 ± 0.12^c^	22.12 ± 0.29^c^
Group 4	28.57 ± 0.29^b^	47.34 ± 0.30^c^	2.96 ± 0.29^c^	24.19 ± 0.32^c^
Group 5	25.42 ± 0.35^d^	56.80 ± 0.37^d^	3.46 ± 0.58^d^	28.57 ± 0.37^d^

LPO – Lipid peroxidation, CAT – catalase, SOD – superoxide dismutase, GSH – reduced glutathione. n = 6 animals in each group. Values are expressed as mean ± SEM. Unpaired ‘t’ test when compared with vehicle control

a *P* < 0.001. One way ANOVA followed by Dunnett’s test when compared with Group 2

b *P* < 0.05,

c *P* < 0.01,

d *P* < 0.001.

The results of our study showed that administration of high fat diet induced significant production of MDA in liver, and administration of Kumbhajatu significantly decreases the MDA production in liver. Kumbhajatu also resulted in a significant increase in the liver CAT, SOD and reduced GSH levels as compared to the control animals, which suggests its antioxidant activity.

## DISCUSSION

Hypercholesterolemia, a high cholesterol diet and oxidative stress increase serum LDL levels resulting in increased risk for development of atherosclerosis.[9] Malondialdehyde a secondary product of lipid peroxidation is a major reactive aldehyde; higher levels can lead to peroxidation of biological membranes.[10] The antioxidant enzymes, mainly superoxide dismutase and catalase are first-line defensive enzymes against free radicals.[11] The qualitative analysis of Kumbhajatu indicated the presence of flavonoids and polyphenols. It is well known that flavonoids and polyphenols are natural antioxidants but have also been reported to significantly increase SOD, glutathione and catalase activities. Further, it was shown that these compounds act as promoters for SOD, glutathione and catalase and cause the expression of SOD, glutathione and catalase.[12] The currently noted elevated levels of SOD, glutathione and catalase with Kumbhajatu could be due to the influence of flavonoids and polyphenols. Lipid peroxidation is a free radical mediated process, which has been accepted to be one of the principle causes of cholesterol-induced diseases, and is mediated by the production of free radical derivatives.[13] Biological membranes are often rich in unsaturated fatty acids and bathed in oxygen-rich metal containing fluid. Therefore, it is not surprising that membrane lipids are susceptible to peroxidative attack.[14] The biochemical mechanisms involved in the development of hypercholesterolemia have long been investigated. MDA, a stable metabolite of the free radical mediated lipid peroxidation cascade, is widely used as marker of lipid peroxidation. Lipid peroxide levels in tissue were found to be significantly elevated in hypercholesterolemic rats.[15] The GSH, catalase, SOD antioxidant system plays a fundamental role in cellular defense against reactive free radicals and other oxidant species. The principal constituents of Lodhra are three alkaloids, viz. loturine, loturidine and collturin which are known to possess free radical scavenging properties.[16] Jatamansi is also known for its antioxidant properties due possibly due to the presence of jatamansone and jatamanshic acid.[17] The antioxidant activity of Kumbhajatu may further be attributed to the high phenolic content of Kumbhi.[18] Shilajit, being an adaptogen, reverses defective electron transport chain reactions. Thus it decreases increased turnover of superoxide anions. The active constituent of shilajit consists of dibenzo-alpha-pyrones and related metabolites, small peptides and fulvic acid. Dibenzopyrones possess the property to augment antioxidant defense and energy, generally restoring normal mitochondrial functions.[19] *C.arborea* contains large amounts of saponins. Most saponins form an insoluble complex with 3β hydroxysteroids, and consequently with cholic acids and cholesterol.[20] To conclude, feed supplementation with Kumbhajatu reduced the hyperlipidemic and oxidative conditions. Kumbhajatu appears to ameliorate hypercholesterolemia probably by decreasing the exogenous cholesterol absorption and increasing the endogenous cholesterol conversion to bile acid, though to know the exact mechanism further studies are needed.
